# CD163+ tumor-associated macrophage is a prognostic biomarker and is associated with therapeutic effect on malignant pleural effusion of lung cancer patients

**DOI:** 10.18632/oncotarget.3547

**Published:** 2015-03-12

**Authors:** Li Yang, Fei Wang, Liping Wang, Lan Huang, Jing Wang, Bin Zhang, Yi Zhang

**Affiliations:** ^1^ Biotherapy Center, The First Affiliated Hospital of Zhengzhou University, Zhengzhou, Henan Province, China; ^2^ School of Life Sciences, Zhengzhou University, Zhengzhou, Henan Province, China; ^3^ Department of Oncology, The First Affiliated Hospital of Zhengzhou University, Zhengzhou, Henan Province, China; ^4^ Department of Respiration, The First Affiliated Hospital of Zhengzhou University, Zhengzhou, Henan Province, China; ^5^ Robert H. Lurie Comprehensive Cancer Center, Department of Medicine-Division of Hematology/Oncology, Northwestern University Feinberg School of Medicine, Chicago IL, USA

**Keywords:** malignant pleural effusion, tumor-associated macrophages, CD163, prognostic biomarker, therapeutic effect

## Abstract

CD163+ tumor-associated macrophages (TAMs) play an important role in the progression of cancer. However, the significance of CD163+ TAMs in malignant pleural effusion (MPE) is still unclear. The aim of this study is to evaluate the prognostic value of CD163+ TAMs in MPE, and the regulatory effect of an immune adjuvant (pseudomonas aeruginosa - mannose-sensitive hemagglutinin, PA-MSHA, which is used for MPE treatment in clinic) on CD163+ TAMs in MPE. Here, we found that the percentage of CD163+ TAMs in MPE was significantly higher than that in non-malignant pleural effusion (*P*<0.001). More importantly, CD163+ TAMs in MPE patients were an independent prognostic factor for progression-free survival. M2-related cytokines were highly expressed in MPE-derived CD163+ TAMs than in MPE-derived CD163− macrophages (*P*<0.05). CD163+ TAMs frequency in MPE patients was obviously reduced after PA-MSHA treatment in clinic (*P*<0.05). After treatment with PA-MSHA, M2 macrophages were re-educated to M1 macrophages *in vitro*. TLR4 blocking antibody inhibited M2 macrophages polarization to M1 macrophages induced by PA-MSHA. These findings highlight that accumulation of CD163+ TAMs in MPE caused by lung cancer is closely correlated with poor prognosis. CD163+ TAMs are associated with therapeutic effect in MPE. PA-MSHA re-educates CD163+ TAMs to M1 macrophages through TLR4-mediated pathway in MPE.

## INTRODUCTION

Malignant pleural effusion (MPE) is a frequent problem faced by clinicians, which is usually associated with cancer-related mortality and morbidity, and reduces the quality of life as well [1, 2]. MPE is a complication that occurs in 30% of lung cancers. It can also occur with other cancers, such as breast cancers and lymphomas. The main goals in the treatment of MPE are the removal of effusion, the improvement in symptoms and the prevention of re-accumulation. Therapeutic thoracentesis and fluid aspiration should be the first medical procedure in the management of MPE which are useful in determining the effects on breathlessness. Thoracentesis has limited effect as a permanent therapeutic approach. Chemotherapy is effective in controlling the production only in non-small cell of lung cancer (NSCLC) patients [3].

The cellular microenvironment of MPE is crucial for the growth, progression and metastasis of malignant tumors [4, 5]. Macrophages that infiltrate tumor tissues are referred as tumor-associated macrophages (TAMs) [6-8]. Macrophage can differentiate into a classically activated phenotype (M1) and an alternatively activated phenotype (M2). M1 macrophages are characterized by the expression of many pro-inflammatory cytokines. Many factors produced by alternatively activated M2 macrophages act in favor of tumor progression [9]. CD163 is a surface receptor on cells of the monocytic lineage [10]. It was initially identified as a scavenger receptor that internalizes hemoglobin/haptaglobin complexes, but also interacts with erythroblasts, distinct pathogens and molecular ligands. CD163+ TAMs are a hallmark of the tumor microenvironment and have been associated with poor prognosis in different types of cancer [11, 12]. In our previous study, CD163+ TAMs can serve as a diagnostic biomarker for MPE. However, the prognostic significance of CD163+ TAMs and their association with therapeutic effect in MPE are still unclear.

In clinic, pseudomonas aeruginosa-mannose-sensitive hemagglutinin (PA-MSHA) has been used to control MPE in patients with lung cancer [13]. PA-MSHA, developed through biological engineering technology based on P. aeruginosa mannose-sensitive hemagglutination pilus vaccine strains, has been successfully used as a protective vaccine [14]. It has been demonstrated that PA-MSHA can trigger naïve immune responses through the activation of multiple immune cells including macrophages [14]. However, the molecular mechanism of PA-MSHA for MPE treatment is still unclear. Furthermore, it is valuable to study whether the effect of PA-MSHA on MPE is associated with CD163+ TAMs.

Therefore, the aim of this study is to evaluate the prognostic value of CD163+ TAMs, and their association with therapeutic effect of MPE, especially the effect of PA-MSHA on CD163+ TAMs in MPE.

## RESULTS

### The level of CD163+ TAMs is increased in MPE

**Figure 1:**
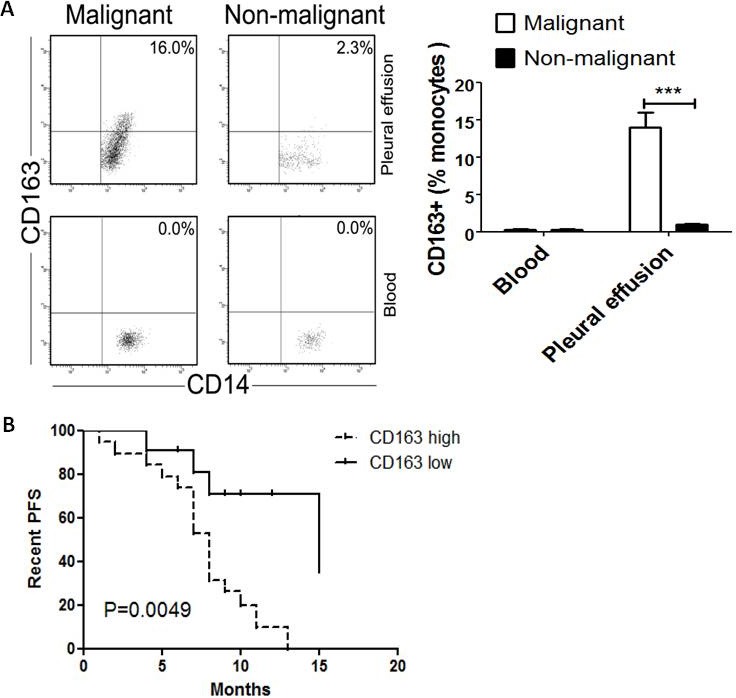
CD163+ TAMs inversely correlate with PFS in MPE patients A, Blood-derived and pleural effusion-derived CD163+CD14+ macrophages were analyzed by flow cytometry in MPE and NMPE patients. One representative analysis from MPE and NMPE cases is shown. Comparison of CD163+ TAMs frequency in blood and pleural effusion from MPE and NMPE patients are presented as histogram. *** = *P*<0.001. B, Kaplan-Meier survival curves for 30 patients with MPE caused by lung cancer (flow cytometric analysis).

M2 macrophages play an important role in the growth and progression of cancers. CD163 has been reported as a specific marker for M2 macrophages [12]. So we detected surface CD163 expression on CD14+ macrophages derived from pleural effusion and peripheral blood in cancer patients or non-cancer patients by flow cytometry, respectively. The results showed that the percentage of CD163+CD14+ cells was significantly higher in MPE than that in non-malignant pleural effusion (NMPE) (*P*<0.001, Figure 1A). On the other hand, CD163 expression was barely detectable in peripheral blood from both cancer and non-cancer patients (Figure 1A). These data indicate that the level of CD163+ TAMs is increased in MPE caused by lung cancer.

### Impact of CD163+ TAMs on survival

Whether CD163+ TAMs are associated with poor prognosis in MPE patients, it is still unclear. Then the impact of CD163+ macrophages on survival was evaluated in MPE patients. For CD163+ macrophages frequency, patients were grouped as “high” or “low” using the respective median (12%) as a cut-off point. The prognostic value of CD163+ macrophages in MPE was evaluated in lung cancer. MPE patients with dense infiltration of CD163+ macrophages had a worse progression-free survival (PFS) (*P*=0.0049, Figure 1B). Therefore, CD163+ TAMs in lung cancer patients with MPE was an independent prognostic factor for PFS.

### High expression of M2-related cytokines in MPE-derived CD163+ TAMs

**Figure 2:**
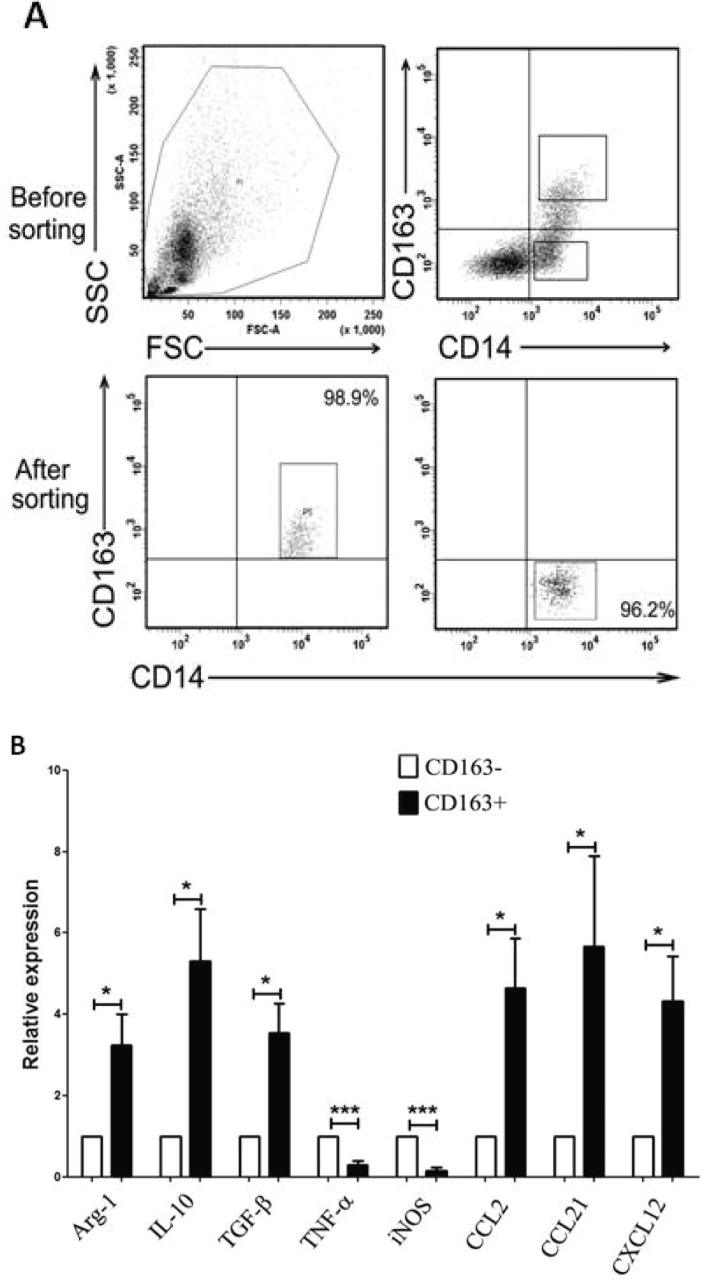
Expression of M1- and M2- related cytokines in CD163+ and CD163− macrophages A, CD163+ and CD163− macrophages were sorted by FACS, respectively. One representative analysis is shown. B, Relative expression level of M1-related cytokines (TNF-α and iNOS) and M2-related cytokines (Aginase-1, IL-10, TGF-β, CCL2, CCL21, CXCL12) in CD163+ and CD163− macrophages was analyzed by real-time PCR. Results are presented as histogram. * = *P*<0.05, *** = *P*<0.001.

The close correlation between CD163+ TAMs and poor prognosis in MPE patients suggests that CD163+ TAMs may serve as a key cell population in the growth and progression of MPE. To further evaluate the function of CD163+ TAMs in MPE, we sorted CD163+ and CD163− cells from MPE by fluorescence activating cell sorter (FACS) for analysis of inflammatory factors and chemokines expression. After sorting, the purity of CD163+ or CD163− macrophages was no less than 95% (Figure 2A). The mRNA expression of anti-inflammatory factors (Aginase-1, IL-10 and TGF-β) and chemokines related to M2 macrophages (CCL2, CCL21 and CXCL12) in CD163+ macrophages was significantly higher than that in CD163− macrophages (*P*<0.05, Figure 2B). Whereas, pro-inflammatory factors of TNF-α and iNOS expression in CD163+ macrophages was lower compared to that in CD163− macrophages (*P*<0.001, Figure 2B). Taken together, high expression of M2-related cytokines in MPE-derived CD163+ macrophages demonstrates that CD163+ TAMs are defined as M2 macrophages in MPE, and closely associated with tumor progression.

### MPE-derived CD163+ TAMs are decreased after treatment with PA-MSHA in clinic

**Figure 3:**
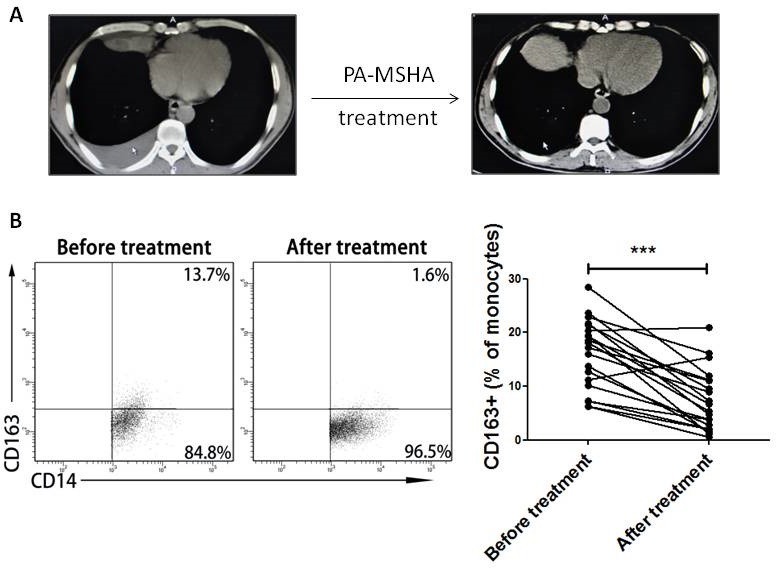
Change of CD163+ TAMs frequency before and after treatment with PA-MSHA in clinic A, Chest CT imaging of one patient with MPE before and after treatment of PA-MSHA in clinic. Arrow shows the location of pleural effusion in CT imaging. B, CD163+ TAMs frequency in MPE before and after treatment with PA-MSHA in clinic was analyzed by flow cytometry. One representative analysis is shown. Comparison of CD163+ TAMs frequency in MPE before and after treatment with PA-MSHA is presented as before-after graph. *** = *P*<0.001.

In clinic, PA-MSHA has been used to treat MPE caused by lung cancer [13]. Within 12 h of PA-MSHA treatment, the volumes of pleural effusion were gradually decreased (Figure 3A). According to the report, PA-MSHA can trigger naïve immune responses through the activation of multiple immune cells including macrophages [14]. In addition, in our study we previously found that CD163+ TAMs frequency in MPE was higher than that in NMPE. So we analyzed the effect of PA-MSHA on CD163+ TAMs in clinic. The percentage of CD163+ TAMs in 30 MPE patients was decreased after treatment with PA-MSHA (*P*<0.001, Figure 3B). The results suggest that the therapeutic effect of PA-MSHA on MPE is closely correlated with CD163+ TAMs.

### M2 macrophages are re-educated to M1 macrophages after PA-MSHA treatment

**Figure 4:**
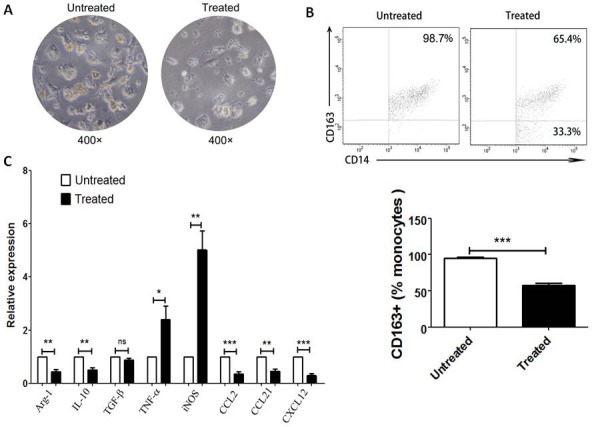
M2 macrophages are re-educated to M1 macrophages after PA-MSHA treatment *in vitro* The cytology (A) and the percentage (B) of CD163+ macrophages was analyzed by microscopy (×400) and by flow cytometry respectively before and after treatment with PA-MSHA *in vitro*. A, One representative analysis is shown. B, The results of CD163+ macrophages frequency before and after PA-MSHA treatment are presented as histogram. C, Relative expression of M1-related cytokines (TNF-α, iNOS) and M2-related cytokines (Aginase-1, IL-10, TGF-β, CCL2, CCL21, CXCL12) in CD163+ macrophages before and after treatment with PA-MSHA was analyzed by real-time PCR. Results are presented as histogram. * = *P*<0.05, ** = *P*<0.01, *** = *P*<0.001, ns = non-significance.

To further investigate the effect of PA-MSHA on CD 163+ macrophages, the cytology and expression of M1- and M2-realated cytokines of CD163+ TAMs before and after treatment with PA-MSHA were analyzed *in vitro*. After treatment with PA-MSHA, CD163+ cells became smaller and turned round (Figure 4A), which are looked as M1 macrophages compared to M2 macrophages. And it was validated that these cells with morphological changes were not apoptosis cells (data not shown). Also, the frequency of purified CD163+ macrophages was decreased after treatment with PA-MSHA *in vitro* (*P*<0.001, Figure 4B). In addition, we found that the mRNA expression of anti-inflammatory factors (Aginase-1, IL-10) and M2-related chemokines (CCL2, CCL21 and CXCL12) in CD163+ macrophages treated with PA-MSHA was lower than that in those cells untreated with PA-MSHA (*P*<0.01, Figure 4C). However, pro-inflammatory factors (TNF-α and iNOS) expression in these cells was increased after treatment with PA-MSHA (*P*<0.05, Figure 4C). All of the results demonstrate that M2 macrophages are re-educated to M1 macrophages induced by PA-MSHA.

### PA-MSHA restores NK cytotoxicity impaired by CD163+ TAMs

**Figure 5:**
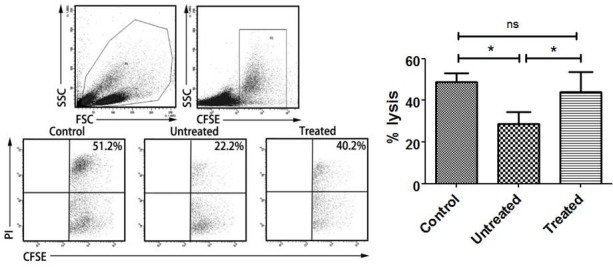
Effect of PA-MSHA on NK cytotoxicity impaired by CD163+ TAMs NK cytotoxicity assay was analyzed by flow cytometry. One representative analysis is shown. The results of NK cytotoxicity assay are presented as histogram. Control = NK cells + K562 cells; Untreated = macrophages + NK cells + K562 cells; Treated = macrophages + NK cells + K562 cells + PA-MSHA. * = *P*<0.05, ns = non-significance.

Then we investigated CD163+ TAMs affected NK cytotoxicity. With co-incubation of CD163+ TAMs, NK cell killing was significantly lower than control (*P*=0.0291, Figure 5), which showed that CD163+ TAMs inhibited NK cells killing. After that, the effect of CD163+ TAMs on NK cytotoxicity affected by PA-MSHA was performed. After treatment with PA-MSHA, NK cytotoxicity was obviously reversed compared to untreated group (*P*=0.0339, Figure 5). Overall, PA-MSHA restores the cytotoxicity of NK cells impaired by CD163+ TAMs.

### Toll-like receptor 4 (TLR4) blocking antibody inhibits M2 macrophages polarization to M1 macrophages induced by PA-MSHA

**Figure 6:**
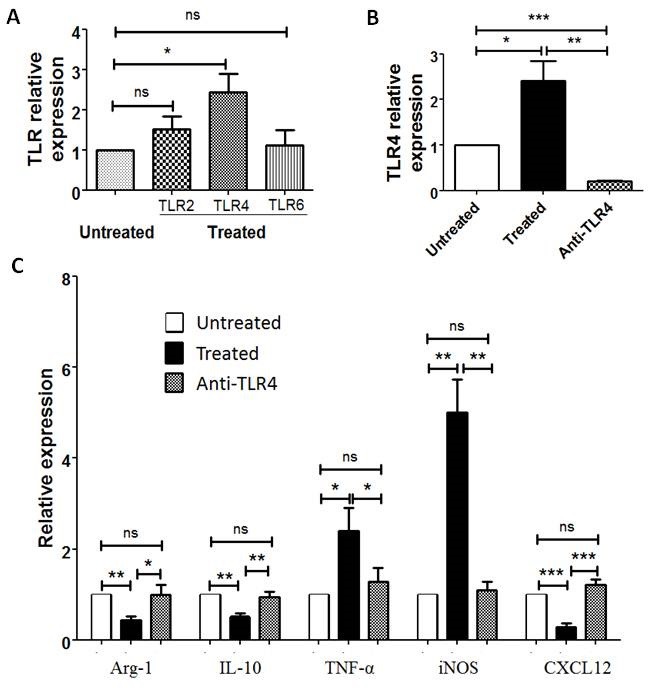
TLR4 blocking antibody inhibits M2 macrophages polarization to M1 macrophages induced by PA-MSHA A, Relative expression of TLR2, TLR4 and TLR6 in CD163+TAMs with PA-MSHA treatment compared to untreated with PA-MSHA *in vitro* was analyzed by real-time PCR. B, TLR4 expression in CD163+ TAMs after using anti-TLR4 blocking antibody was analyzed by real-time PCR. C, Relative expression of Aginase-1, IL-10, TNF-α, iNOS, CXCL12 in CD163+ macrophages with and without using TLR4 blocking antibody was analyzed by real-time PCR. Untreated = untreated with any PA-MSHA and TLR4 blocking antibody; Treated = treated with PA-MSHA; Anti-TLR4 = treated with PA-MSHA and TLR4 blocking antibody. Results are presented as histogram. * = *P*<0.05, ** = *P*<0.01, *** = *P*<0.001, ns = non-significance.

The mechanism underlying the role of PA-MSHA in enhancing immunity primarily relies on PA-MSHA composition: MSHA fimbriae can activate pattern recognition receptors, including TLR4 [15], and activate numerous immune cells [16-18]. In our study, we investigated TLR2, TLR4 and TLR6 expression in CD163+ TAMs treated or untreated with PA-MSHA *in vitro*. After PA-MSHA treatment, the mRNA expression of TLR4 in CD163+ macrophages was obviously increased (*P*=0.0264, Figure 6A), whereas TLR2 and TLR6 mRNA expression in CD163+ macrophages was not significant difference compared to untreated with PA-MSHA (*P*>0.05, Figure 6A). To determine whether the TLR4 signaling pathway is important for PA-MSHA induced re-education of CD163+ macrophages, CD163+ macrophages were treated with TLR4 blocking antibody prior to treatment with PA-MSHA for 6h *in vitro*. The expression of TLR4 in these macrophages treated with anti-TLR4 blocking antibody was decreased compared to treated group (*P*=0.0037) and untreated group (*P*<0.001, Figure 6B). Real-time PCR analyses showed that there were significant differences of cytokines and chemokines expression in CD163+ macrophages treated with anti-TLR4 blocking antibody compared to treated group (*P*<0.05, Figure 6C). Whereas anti-TLR4 blocking antibody restored the expression of M1- and M2- related cytokines in these macrophages to the level of untreated group (*P*>0.05, Figure 6C). Collectively, the results suggest that the TLR4 signaling pathway is important for macrophages polarization induced by PA-MSHA in MPE.

## DISCUSSION

Macrophages are activated differentially according to the stimuli provided: M1 anti-tumorigenic macrophages are defined as classically activated subset, and M2 pro-tumorigenic macrophages (also known as TAMs) are defined as alternatively activated subset [19-21]. TAMs are prominently involved with cancer initiation, progression and metastasis, facilitating angiogenesis, matrix breakdown, and tumor cell-motility [22-24]. Also, TAMs likely play important functions in human tumors as their number and density positively correlate with a poor prognosis [25].

CD163 is a scavenger receptor on cells of the monocytic lineage, with weak expression on peripheral blood monocytes and abundant expression on the majority of tumor-derived macrophages [26-28]. In lung tumor sections, the number of CD163+CD14+ cells is higher in malignant lesions compared to begin lesions and correlates with histological grading of malignancy [29, 30]. Behnes et al [31] found that nearly all macrophages in papillary renal cell carcinoma (RCC) type II expressed CD163, whereas in type I papillary RCC, less than 30% of macrophages expressed CD163, which might explain why the prognosis of papillary RCC type II was worse than that of type I. Our study showed that the frequency of CD163+ macrophages was higher in MPE than those in NMPE. In addition, in this study high expression of M2-related cytokines in CD163+CD14+ macrophages suggests that these macrophages are considered as M2 protumoral macrophages, which is in accordance with concept of these papers.

Furthermore, we investigated the impact of CD163+ TAMs on survival of MPE patients. The results showed that CD163+ TAMs in lung cancer patients with MPE is an independent prognostic factor for PFS. Other studies also proved the presence of TAMs in tumor stroma as a prognostic marker for cancer patients [32-34]. Medrek et al [32] found that some CD163+ areas lacked CD68 expression, suggesting that CD163 could be used as a general anti-inflammatory myeloid marker with prognostic impact. Shabo et al [33] also reported that CD163 expression in rectal cancer cells was related to early local recurrence, shorter survival time and reduced apoptosis. In clinic, PA-MSHA was used for MPE treatment. With the treatment of PA-MSHA for MPE, the level of CD163+ TAMs was decreased in our study, which demonstrates that CD163+ TAMs may serve as a potential biomarker for therapeutic effect of MPE. This concept was further proved *in vitro* in this study. So far there are no other studies focused on factors associated with therapeutic effect of MPE.

Early studies indicated that PA-MSHA can fight against liver cancer, gastric cancer, and breast cancer cell lines [14, 35-37]. PA-MSHA, developed through biological engineering technology based on P. aeruginosa mannose-sensitive hemagglutination pilus vaccine strains, has been successfully used as a protective vaccine. The mechanism underlying the role of PA-MSHA in enhancing immunity primarily relies on PA-MSHA composition: MSHA fimbriae can activate pattern recognition receptors including TLR4 [15], and activate numerous immune cells, such as dendritic cells, macrophages, T cells and NK cells, to assist in the reconstruction of immune surveillance and defense [16-18]. PA-MSHA can also activate the immune response through TLRs-mediated signal transduction. However, whether PA-MSHA is affected on CD163+ TAMs is still unclear. Therefore, we further evaluated the effect of PA-MSHA on CD163+ TAMs and its possible molecular mechanism.

In this study, the results suggest that M2 macrophages are re-educated to M1 macrophages induced by PA-MSHA *in vitro*. So we hypothesize that the decrease of pleural effusion after PA-MSHA treatment in clinic might correlate with M2 macrophages re-education to M1 macrophages. M1 macrophages are acted as anti-tumorigenic macrophages, which are characterized by the expression of many pro-inflammatory cytokines, such as TNF-α, IL-1β, IFN-γ and so on [9]. These pro-inflammatory cytokines derived from M1 macrophages might have direct effect on tumor cells apoptosis in MPE, which are due to the decrease of pleural effusion after PA-MSHA treatment.

Members of the TLR family play key roles in both innate and adaptive immune responses [38]. TLR proteins enable host to recognize a large number of pathogen-associated molecular patterns such as bacterial lipopolysaccharides, viral RNA, CPG-containing DNA, and flagellin, among others. TLRs are involved in the development of many pathological conditions including infectious diseases, tissue damage, autoimmune and neurodegenerative diseases and cancer. In our study, PA-MSHA treatment enhanced TLR4 expression which is similar to a report from Zhu et al [14]. TLR2 and TLR6 expression on CD163+ macrophages after PA-MSHA treatment *in vitro* was not significant increased. Anti-TLR4 blocking antibody restored the expression of M1- and M2- related cytokines in these macrophages treated with PA-MSHA. Anti-TLR4 blocking antibody inhibits M2 macrophages polarization to M1 macrophages induced by PA-MSHA. The results demonstrate that the mechanism of PA-MSHA in enhancing immunity primarily relies on activation of TLR4.

Taken together, significant accumulation of CD163+ TAMs in MPE caused by lung cancer is closely correlated with poor prognosis. CD163+ TAMs are associated with the therapeutic effect of MPE. PA-MSHA re-educates CD163+ TAMs (M2 macrophages) to M1 macrophages in MPE via TLR4-mediated pathway.

## MATERIALS AND METHODS

### Patients

Sixty patients with pleural effusion were recruited at The First Affiliated Hospital of Zhengzhou University from May 2011 to December 2013. Pleural effusion and peripheral blood were collected from 30 patients with lung cancer and 30 NMPE patients. In addition, another 30 patients with MPE treated with PA-MSHA (Beijing Wanter Bio-pharmaceutical Co.) were also recruited from December 2011 to December 2013. All samples were obtained with the approval from Ethics Committee of the hospital. Inclusion criteria of MPE were lung cancer, proven by histopathological examination of lung biopsy material and an age >18 years, without diseases of immune system. Inclusion criteria of NMPE were pneumonia, tuberculosis and heart failure / hypoproteinemia. Exclusion criteria of NMPE were a history of malignant disease within the last five years and solid organ or bone marrow transplantation.

### Flow cytometric analysis

Mononuclear cells from pleural effusion or peripheral blood were isolated by Ficoll-Hypaque (Huajing Biology Co., Shanghai) density gradient centrifugation. 1×10^5^ cells were stained with APC-Cy7 labeled anti-human CD14 (Biolegend) and PE labeled anti-human CD163 (Biolegend) antibodies. Dead cells were stained using 7-AAD (BD Biosciences). After incubation for 15 min on ice in the darkness, the cells were analyzed by FACSCanton II (BD).

To investigate the effect of PA-MSHA on CD163+ macrophages, the percentages of CD163+ macrophages in MPE before and after treatment of PA-MSHA in clinic and *in vitro* were analyzed by flow cytometry as above method, respectively.

### Cell isolation

CD163+CD14+ and CD163−CD14+ populations were sorted from mononuclear cells derived from MPE using Moflo XDP (Beckman) (n=6). In brief, cell clumps were removed by passing cell suspensions through 40 mm Cell Strainers (BD Biosciences). 1×10^8^ mononuclear cells were stained with 20 μl of anti-human CD163, CD14 and 7-AAD antibodies (Biolegend) respectively. Then, cells were incubated in the dark for 15 min at 4 ^°^C. Cells were resuspended with 1 ml of normal saline for sorting. The purities of sorted CD163+CD14+ and CD163−CD14+ cells were analyzed by FACS.

### RNA extraction and real-time PCR analysis

Total RNA was extracted from purified CD163+CD14+ and CD163−CD14+ cells using Trizol Reagent (Sigma Aldrich). Then reverse transcription was performed by using cDNA synthesis Kit (TaKaRa) according to the manufacturer' instructions. cDNA was used as the template for real-time PCR using SYBR Premix ExTaq II (TaKaRa) on Stratagene Mx3005P (Agilent Technologies). The sequences of primers for human Arginase-1, IL-10, TGF-β, TNF-α, iNOS, CCL2, CCL21 and CXCL12 were listed in Table 1. Samples were amplified using the following conditions: 40 cycles of 95^°^C/30sec, 95^°^C/5sec, 60^°^C/30sec. The abundance of mRNA for each gene of interest was normalized to GAPDH mRNA.

**Table 1:** The sequences of primers used for quantitative real-time PCR

No.	Gene	Sense Primer	Antisense Primer
1	Arginase-1	5′TCCCTGTATATCTGCCAAGGATATT3′	5′TTCCTAGTCTGTCCACTTCAGTCAT3′
2	IL-10	5′TTTAAGGGTTACCTGGGTTGC3′	5′TTGATGTCTGGGTCTTGGTTC3′
3	TGF-β	5′GCCAGAGTGGTTATCTTTTGATG3′	5′AGTGTGTTATCCCTGCTGTCAC3′
4	IFN-γ	5′ATTCGGTAACTGACTTGAATGTCC3′	5′CTCTTCGACCTCGAAACAGC3′
5	TNF-α	5′CTGTAGCCCATGTTGTAGCAAAC3′	5′GCTGGTTATCTCTCAGCTCCAC3′
6	iNOS	5′GCCAAGCTGAAATTGAATGAGGA3′	5′ TTCTGTGCCGGCAGCTTTAAC3′
7	CCL2	5′CAGCCAGATGCAATCAATGCC3′	5′TGGAATCCTGAACCCACTTCT3′
8	CCL21	5′GTTGCCTCAAGTACAGCCAAA3′	5′AGAACAGGATAGCTGGGATGG3′
9	CXCL12	5′ATTCTCAACACTCCAAACTGTGC3′	5′ACTTTAGCTTCGGGTCAATGC3′
10	TLR2	5′ TTATCCAGCACACGAATACACAG3′	5′AGGCATCTGGTAGAGTCATCAA3′
11	TLR4	5′TACAAAATCCCCGACAACCTCC3′	5′GCTGCCTAAATGCCTCAGGG3′
12	TLR6	5′TTCTCCGACGGAAATGAATTTGC3′	5′CAGCGGTAGGTCTTTTGGAAC3′

In addition, the mRNA expression of Arginase-1, IL-10, TGF-β, TNF-α, iNOS, CCL2, CCL21, CXCL12, TLR2, TLR4 and TLR6 in CD163+CD14+ cells before and after treatment of PA-MSHA was analyzed by real-time PCR as above method. The sequences of primers were showed in Table 1. Before and after usage of blockade antibody of TLR4, the mRNA expression of Arginase-1, IL-10, TNF-α, iNOS, CXCL12 in CD163+ macrophages was also analyzed by real-time PCR.

### PA-MSHA treatment *in vitro*

CD163+ macrophages sorted from MPE (n=6) were cultured in RPMI 1640 medium with 10% FBS *in vitro*. After treatment with PA-MSHA at a final concentration of 90 U/ml for 6 h, CD163+ macrophages were analyzed for cytology, flow cytometry and expression of M1- and M2-related genes.

### Cytological analysis

To evaluate the effect of PA-MSHA on CD163+ macrophages, the cytology of CD163+ macrophages before and after treatment of PA-MSHA was analyzed by microscopy (400×).

### NK cell cytotoxicity assay

Whether PA-MSHA has influenced on NK cytotoxicity impaired by CD163+ macrophages, we further analyzed. A flow cytometry-based assay was used to analyze NK cell cytotoxicity. K562 (human erythroleukemia cell line) tumor cells, which are known to be sensitive target cells for NK cell lysis, were included in the assays. After treatment with PA-MSHA for 6 h, 1×10^5^ CD163+ macrophages were collected and washed twice, then co-incubated with 1×10^5^ NK cells for 24 h at 37 °C. Then CD163+ macrophages and NK cells were co-incubated with 1×10^4^ CFSE-labeled K562 cells for 5 h at 37 °C. Cells were washed and stained with PI for 2 min. Cells were analyzed by a FACSCantoII flow cytometer, and the percentage of K562 cells undergoing apoptosis was determined. K562 cells treated with NK cells alone in the absence of macrophages were used as a control.

### Anti-TLR4 blocking antibody array

1×10^5^ purified CD163+ TAMs were treated with 10 mg/ml of TLR4 blocking antibody (Biolegend, USA) for 2 h. Then PA-MSHA was added to the wells at a final concentration of 90 U/ml for 6 h. CD163+ TAMs treated with PA-MSHA were used as treated group, CD163+ TAMs untreated with PA-MSHA and anti-TLR4 blocking antibody were used as untreated group. Lastly, macrophages-related cytokines expression in these cells was analyzed by real-time PCR.

### Statistical analysis

All Statistical analyses were performed using the Statistical Program for Social Sciences (SPSS) 17.0 software. Results were analyzed for statistical significance with paired t test. Kaplan-Meier analysis and log rank tests were used to illustrate differences in PFS according to CD163 expression. P value less than 5% was considered as statistically significant.
